# Artificial intelligence-powered smartphone application, AICaries, improves at-home dental caries screening in children: Moderated and unmoderated usability test

**DOI:** 10.1371/journal.pdig.0000046

**Published:** 2022-06-02

**Authors:** Nisreen Al-Jallad, Oriana Ly-Mapes, Peirong Hao, Jinlong Ruan, Ashwin Ramesh, Jiebo Luo, Tong Tong Wu, Timothy Dye, Noha Rashwan, Johana Ren, Hoonji Jang, Luis Mendez, Nora Alomeir, Sherita Bullock, Kevin Fiscella, Jin Xiao

**Affiliations:** 1 Eastman Institute for Oral Health, University of Rochester Medical Center, Rochester, NY, United States of America,; 2 Department of Computer Science, University of Rochester, United States of America,; 3 Department of Biostatistics and computational biology, University of Rochester Medical Center, Rochester, United States of America,; 4 Department of Obstetrics and Gynecology, University of Rochester Medical Center, Rochester, United States of America,; 5 University of Rochester, United States of America,; 6 Temple University School of Dentistry, Pennsylvania, United States of America,; 7 Healthy Baby Network, Rochester, United States of America,; 8 Department of Family Medicine, University of Rochester Medical Center, Rochester, NY, United States of America

## Abstract

Early Childhood Caries (ECC) is the most common childhood disease worldwide and a health disparity among underserved children. ECC is preventable and reversible if detected early. However, many children from low-income families encounter barriers to dental care. An at-home caries detection technology could potentially improve access to dental care regardless of patients’ economic status and address the overwhelming prevalence of ECC. Our team has developed a smartphone application (app), AICaries, that uses artificial intelligence (AI)-powered technology to detect caries using children’s teeth photos. We used mixed methods to assess the acceptance, usability, and feasibility of the AICaries app among underserved parent-child dyads. We conducted moderated usability testing (Step 1) with ten parent-child dyads using “Think-aloud” methods to assess the flow and functionality of the app and analyze the data to refine the app and procedures. Next, we conducted unmoderated field testing (Step 2) with 32 parent-child dyads to test the app within their natural environment (home) over two weeks. We administered the System Usability Scale (SUS) and conducted semi-structured individual interviews with parents and conducted thematic analyses. AICaries app received a 78.4 SUS score from the participants, indicating an excellent acceptance. Notably, the majority (78.5%) of parent-taken photos of children’s teeth were satisfactory in quality for detection of caries using the AI app. Parents suggested using community health workers to provide training to parents needing assistance in taking high quality photos of their young child’s teeth. Perceived benefits from using the AICaries app include convenient at-home caries screening, informative on caries risk and education, and engaging family members. Data from this study support future clinical trial that evaluates the real-world impact of using this innovative smartphone app on early detection and prevention of ECC among low-income children.

## Author summary

Early Childhood Caries (ECC) is the most common childhood disease worldwide and a health disparity among underserved children. ECC is preventable and reversible if detected early. However, many children from low-income families encounter barriers to dental care. An at-home caries detection technology could potentially improve access to dental care regardless of patients’ economic status and address the overwhelming prevalence of ECC. Our team has developed a smartphone application (app), AICaries, that uses artificial intelligence (AI)-powered technology to detect caries using children’s teeth photos. Using AICaries, parents can use their regular smartphones to take photos of their children’s teeth and detect ECC aided by AICaries, enabling them to actively seek treatment for their children at early and reversible stages. Using AICaries, parents can also obtain essential knowledge on reducing their children’s caries risk. The current study evaluated the usability, acceptability, and feasibility of AICaries. Our study results indicated the AICaries app is well-accepted among the participating parents.

## Introduction

Early childhood caries (ECC), the single most common chronic childhood disease, disproportionately afflicts underprivileged preschool children [1,2]. The onset and progression of ECC are “rapid,” “aggressive,” “painful,” “recurrent,” and frequently require total oral rehabilitation under general anesthesia [3]. ECC consequences include a higher risk of new carious lesions, increased treatment costs and time, loss of school days, diminished ability to learn, diminished oral health-related quality of life, hospitalizations, and emergency room visits [4]. It is also a costly disease to treat and constitutes a significant challenge for public health systems [2].

As a multifactorial disease, ECC initiates by microbial fermentation of carbohydrates in dental plaque accumulating on tooth surfaces [5]. The persistent acidic environment in dental plaque leads to the demineralization of tooth hard surfaces, which leads to the eventual development of dental caries [5]. A unique characteristic of dental caries progression is the early reversible stage, at which point decalcified white spots could be re-mineralized with adequate measures, such as proper oral hygiene practice, change from a high sugar diet to a low sugar diet, application of topical fluoride, etc. However, white spots and early-stage caries in early life are often not recognized in a home environment, therefore this critical window is often missed. A calling for at-home caries screening in children via mDentistry tools, such as smartphone app, is urgently needed, particularly among the population facing limited access to dental care [6].

Smartphone applications (apps) have been successfully developed and utilized to assist people in managing health behaviors and conditions [7], such as smoking cessation, weight loss, medication adherence, and Parkinson’s diseases progression monitoring [7,8]. With 77% of Americans owning a smartphone [9], a smartphone app represents a suitable and innovative way of providing oral health interventions among young children and their parents. Current oral health smartphone apps, however, are limited in scope and audience. Most are designed for use by dental professionals to assess caries risk or trauma management, but not for the public, i.e., for users to assess their own risk and/or support users’ self-management of caries risk factors. No apps to our knowledge address the needs of, low-income and minority children who are disproportionately affected by ECC and have limited access to oral health care [10].

Teledentistry is broadly defined by Mariño and Ghanim as “the application of a variety of information and communications technologies (ICT) to facilitate oral health care for geographically distant patients and/or practitioners [11]. Teledentistry holds promise for improving access to care, especially among disadvantaged children [10]. It provides remote oral health care consultation and diagnoses dental caries through images taken by trained professors. However, the diagnostic process of Teledentistry requires dentists to review the photos personally. In addition, some areas lack the infrastructure to support Teledentistry. Artificial intelligence (AI) represents an emerging adjunct to Teledentistry. Although AI is currently used to aid imaging recognition to improve disease diagnosis in many medical fields, including oncology, ophthalmology, radiology, etc. [12–15], AI has not been developed in dentistry for remote caries detection for underserved patients with limited access to oral health care.

To address this gap, we have developed a patient-friendly smartphone app coupled with AI-powered caries detection that holds promise in facilitating early clinical confirmation and treatment of ECC [16]. In this study, we employed a community-based participatory research strategy to test the usability of the AI-powered smartphone app, AICaries, among parents/caregivers for dental caries detection in their children.

## Materials and methods

### The AICaries app components and functional flow

**Fig 1.**
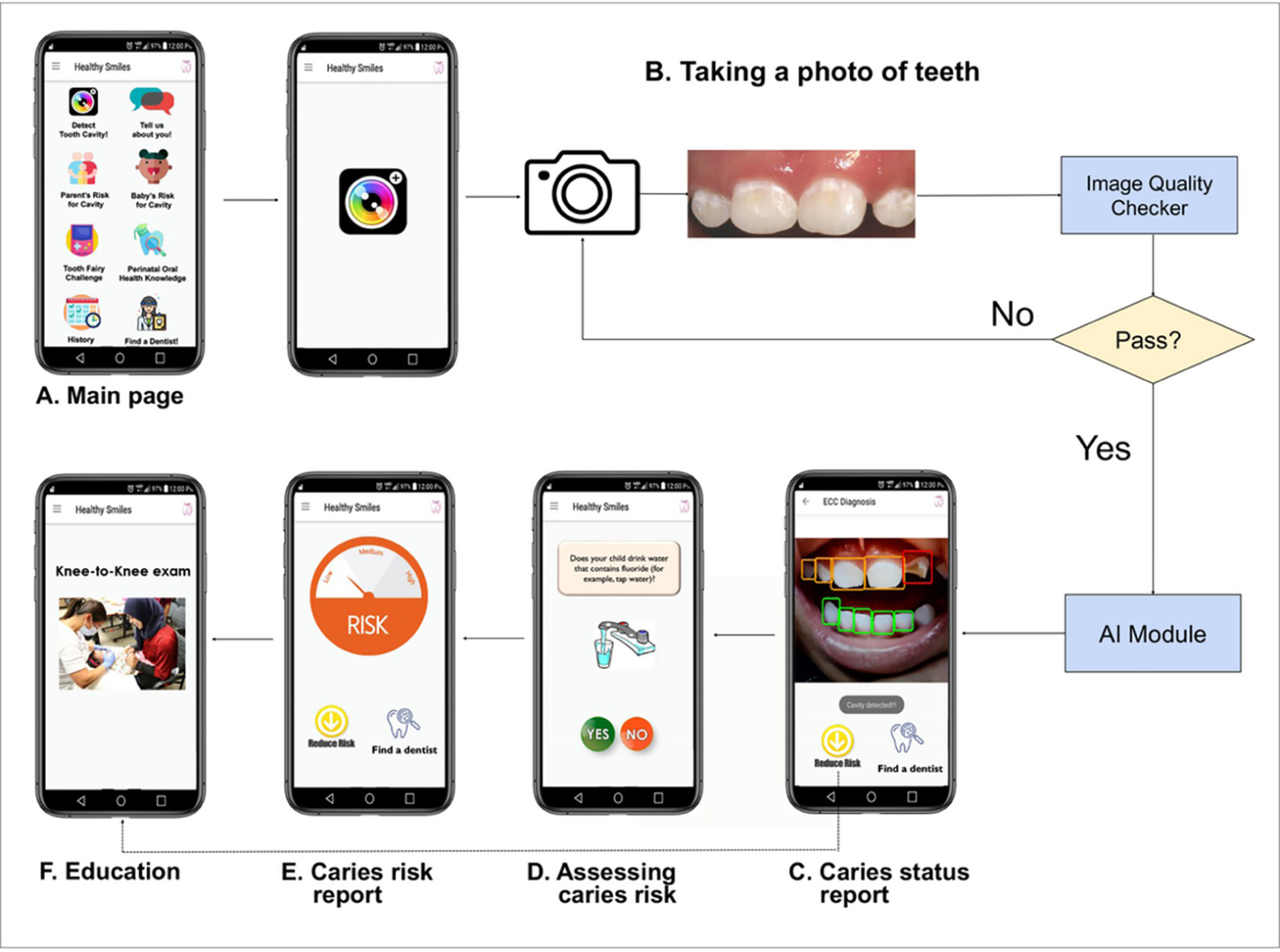
AICaries smartphone app functional flow. A. AICaries app main page. B. To attract app use, users will start with taking teeth photos for caries detection assessment. The teeth photo will go through an Image Quality Checker. Image that passes quality check will get to the next step, “AI-Powered caries detection”. If the image fails the quality check, the app will instruct users to retake teeth photos until desired quality is achieved. C. The app will then generate a Caries Status Report for each tooth. On the Caries Status Report interface, users will make choices for the next step, such as Assess Caries Risk (D) using a previously developed risk assessment system. Users will receive their caries risk as Low, Medium, or High (E). On the Caries Status Report interface (C), user could also choose “Reduce Risk” to access Perinatal Oral Health Education (F) or click “Find a dentist” for dental clinic information. Upper right corner icon links to the app main page. https://doi.org/10.1371/journal.pdig.0000046.g001

Our previous collaborative work by experts in AI imaging recognition, oral health, and mobile health (mHealth) led to the prototype of a patient-friendly smartphone app coupled with AI-powered caries detection. This AICaries app prototype offers a) AI-powered caries detection using photos of children’s teeth taken by the parents’ smartphones, b) interactive caries risk assessment, and c) personalized education on reducing children’s ECC risk. Fig 1 demonstrates the AICaries app components and app functional flow.

To promote app engagement, users start by taking images of their teeth with their smartphone’s camera for caries detection assessment. The photo is processed through an Image Quality Checker that assesses the blurriness, darkness, and brightness of the image (Fig 1B). Images that pass quality check processed to the next step, “AI-Powered caries detection”. If the image fails the quality check, the app instructs users to retake teeth photos until satisfactory quality is achieved. The app then generates a Caries Status Report for each tooth (Fig 1C). On the Caries Status Report interface, users make choices for the next step, such as Assess Caries Risk (Fig 1D) using a previously developed risk assessment system. Users receive their caries risk as Low, Medium, or High (Fig 1E). On the Caries Status Report interface (Fig 1C), user can choose “Reduce Risk” to access Perinatal Oral Health Education (Fig 1F) or click “Find a dentist” for dental clinic information. The performance of AICaries in caries detection was reported previously [17].

### Overview of usability study

**Fig 2.**
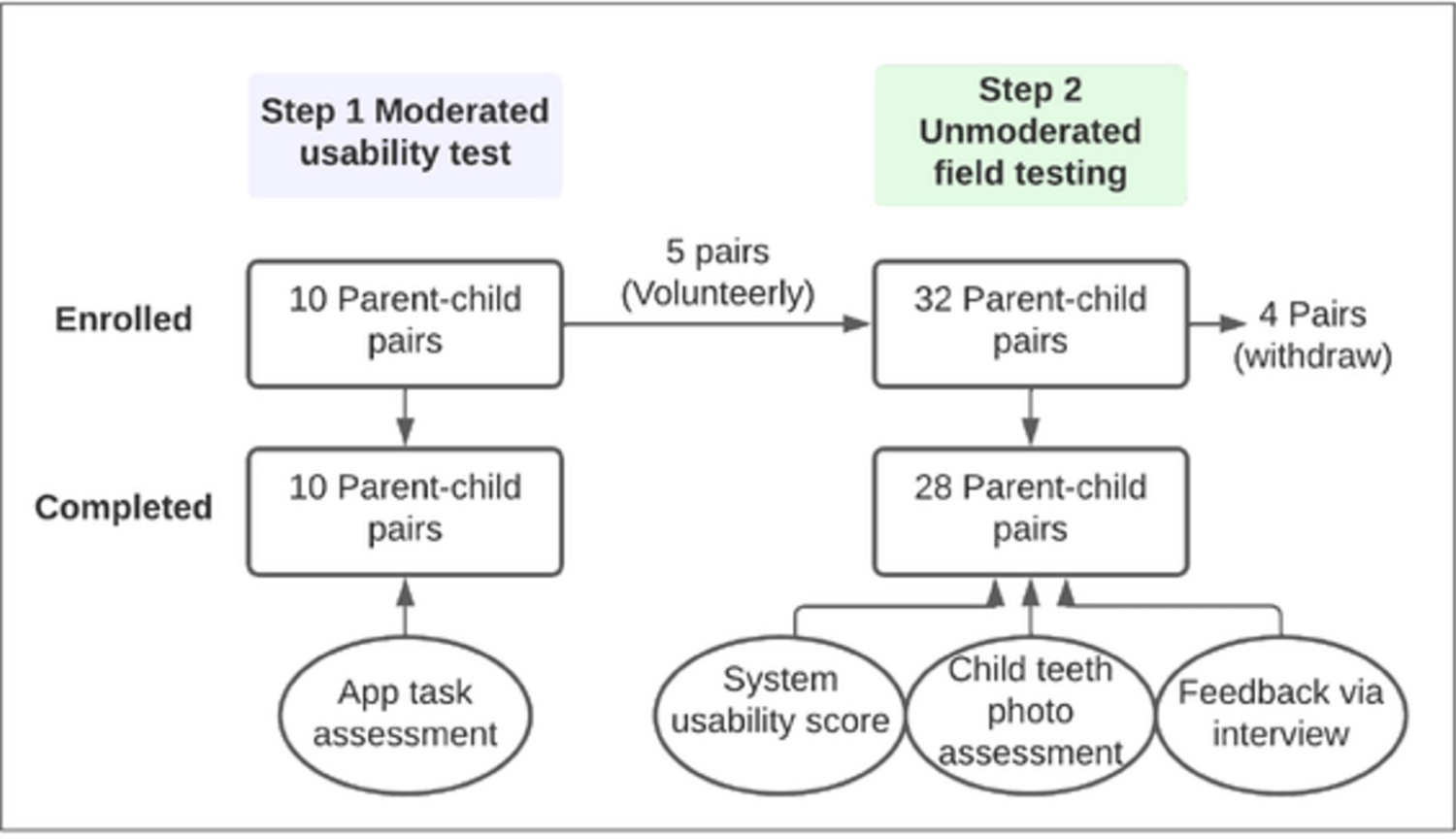
Study flow. https://doi.org/10.1371/journal.pdig.0000046.g002

The research ethics application has been reviewed and approved by the University of Rochester Research Subject Review Board (#5772, 3953, and 5949). In the current study, we tested the usability of AICaries smartphone app in two steps. The detailed study protocol was published previously [16]. Step 1 was the moderated usability test where participants tested the app on a study smartphone, with the assistance from study team; Step 2 was the unmoderated fielding testing where AICaries was installed on participants’ smartphone and tested at-home by participants. Step 1 refined the app based on the participant’s feedback. Step 2 used a mixed methods to obtain the feedback and assess the usability of the app component, app flow, and whether parents can take diagnostic photos of children’s teeth on their own in their natural environment [16]. The study flow seen Fig 2.

### Study participants

A total of 37 parents and 37 children met the eligibility criteria were enrolled in the study. The face-to-face recruitment at the following two sites: the University of Rochester Medical Center (URMC), Eastman Institute for Oral Health (EIOH) Perinatal Dental Clinic and Highland Family Medicine (HFM). The URMC-EIOH Perinatal Dental Clinic serves socioeconomically disadvantaged pregnant women, women postpartum, and young children eligible for State-supported Medicaid type of insurance. The URMC-HFM is one of the largest family medicine residency teaching practices in Rochester, NY, providing comprehensive health care to adults and children from birth through childhood.

Ten parents-child dyads participated the step 1 moderated usability test, 32 parent-child dyads participated Step 2 unmoderated field testing and 28 pairs completed the study. Among the 32 dyads, 5 of them completed Step 1 test and then chose to participate the Step 2 testing.

### Eligibility criteria

The eligibility criteria were a) parents 18 years old or older, who have young children between 1 and 5 years, b) eligible for state-supported Medicaid type of insurance (note, we are using the insurance eligibility to select low-income participants), c) English speaking (consent was written in English and App was in English Language), d) able to provide signed and dated informed consent, and e) have an Android smartphone that could install the AICaries app. Children with orofacial deformity (cleft lip, cleft palate, oral-pharyngeal mass) or Down syndrome or other developmental disabilities were excluded.

### AICaries app task Assessment in Step 1 moderated usability test

We videotaped and immediately reviewed each session using Instant Data Analysis [18] with attention to the photo image quality. We defined key usability tasks necessary to successfully use the app. This included navigation of the app interface, accessing and completing the American Dental Association risk assessment, and notably, taking usable photographs of their child’s front teeth using the photo-taking interface. We ranked each task as 1) critical (required assistance to proceed), 2) severe (major delay and/or frustration), or 3) cosmetic (minor) and annotated to the exact interface/task. Based on rankings, the team discussed changes in the instructional video, app design, and study procedures. We iteratively incorporated changes into subsequent testing sessions. We reached data saturation [19] (no new suggested change were) after conducting 10 individual usability tests.

### Assessment in Step 2 unmoderated field testing

Unmoderated field testing allows the end-users to interact with the AICaries app in their natural environment. We used the same recruitment and eligibility criteria while recruiting parents with a range in education, ages, and smartphone proficiency. Following informed consent, including permission for remote monitoring of the app, the participants downloaded the AICaries app to their smartphones. Over two weeks, text messages sent by the study team reminded participants to take photos of their child’s front teeth. After two weeks, we administered in-person evaluation of System Usability Scale (SUS) via a questionnaire. The SUS instrument [20–22] is widely adopted in business and technology industries and mHealth fields to measure and quantify the perception of product and service usability. It consists of a 10 item questionnaire with five response options for each item from Strongly agree to Strongly disagree [23]. The statements relate to a range of aspects of system use, such as complexity, ease-of-use, and learnability. Each participant’s responses are then scored, providing an overall SUS score between 0 and 100 [24]. A SUS score above 68 indicates above-average usability; a score above 80.3 indicates excellent usability of the AICaries app. Each participant was interviewed to assess their perception of the challenges of using AICaries app, benefits, and suggestions for further improvement. A semi-structured interview guide, detailed in the study protocol [16]. Interview sessions were audio-recorded, transcribed, and analyzed qualitatively for thematic content. A trained dentist assessed the number and quality of images taken.

### Data analysis

For the qualitative data, the transcribed data for Step 1 usability test and Step 2 field testing were coded with predetermined open codes. Thematic content was further analyzed based on participant and team suggestions for addressing barriers to completing a key task related to the use of the app.

For Step 2 field testing, we collected baseline demographic information and participant prior experience with smartphone aps. SUS score stratified by gender, education level, employment status and previous experience of taking teeth photo with phone and compared between above-mentioned categories using Mann-Whitney U test after testing the data normality. Multiple linear regression was conducted to assess the potential association of factors (gender, race, education, previous experience of taking teeth photo) and SUS score. We further assessed quantitative app usage metrics, i.e., time between screens, frequency of use, and numbers/quality (whether photos could be used for clinical diagnosis for dental caries) of images taken. The diagnostic images should include all anatomic structures of the teeth of interest, not be covered by the anatomic structure of other teeth or soft tissue tissues, such as lip or cheek. All statistical tests were two-sided with a significant level of 5%. SPSS IBM was used for statistical analyses.

## Results

### Participant’s characteristics

In step 1, we enrolled total of 10 parents who were 90% female, 20% Black or African American, 60% White, and 20% other or mixed races. We enrolled 32 parents in step 2. The sample was predominantly female and racially diverse (Table 1). Significantly, over 90% of the participant parents reported prior use of medical/health related applications, whereas, none of the participating parents reported experience of using dental care related applications. Interestingly, approximately 60% of the parents reported they had previously tried taking teeth photos with their cell phones.

### AICaries ease of use

**Fig 3.**
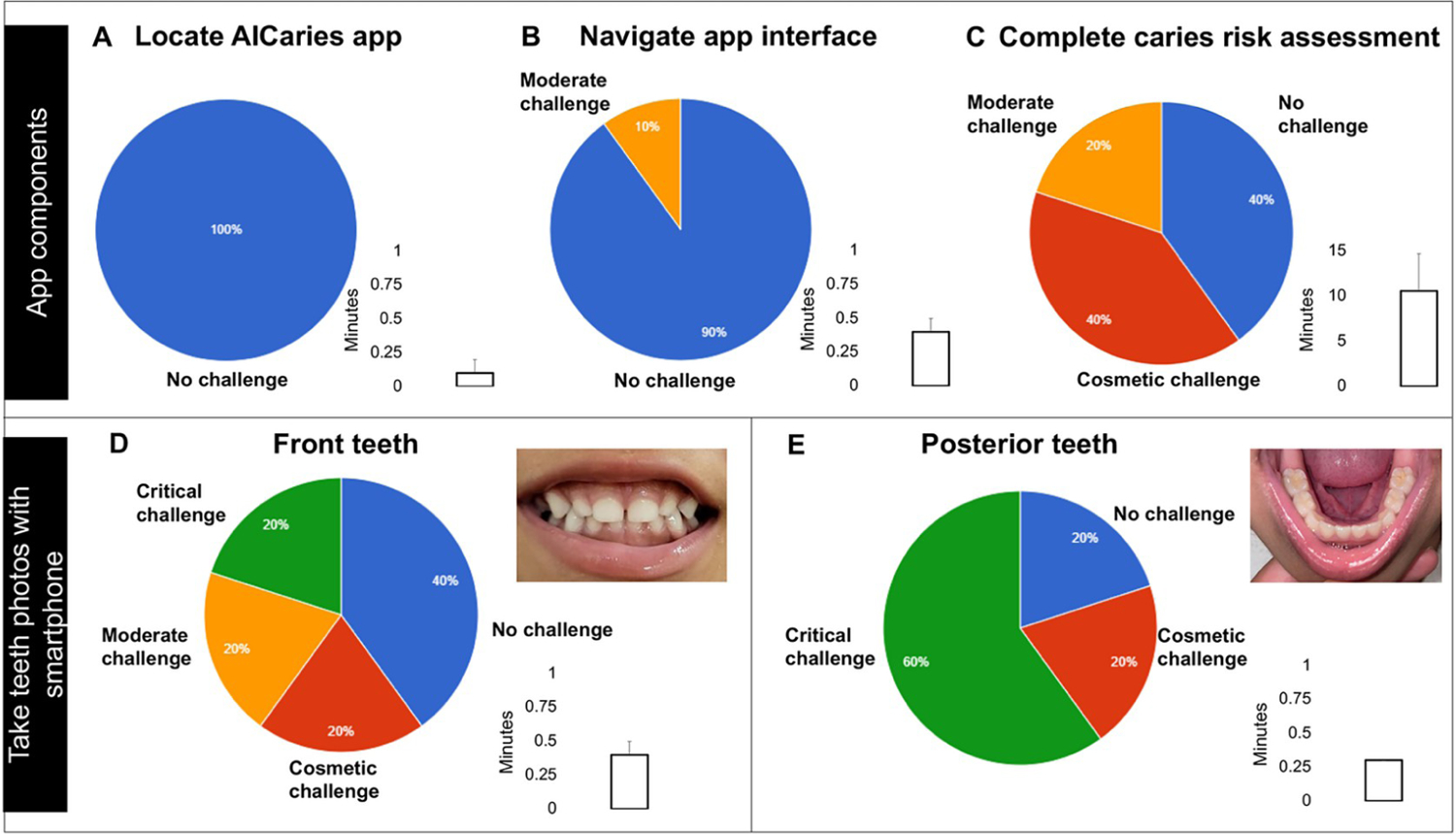
Task assessment and completion time in Step 1 moderated usability test. A total of 7 tasks was assessed. (A) All participating parents (n = 10) located the app icon on the study cellphone with no challenge. (B) 90% of them could navigate the app interface with no challenges. (C) In the caries risk assessment, 40% of the parents completed the risk assessment without challenges, 40% with cosmetic challenges, and 20% with moderate challenges. (D) For taking photos using the AICaries app, 40% of the parents took photos of their children’s front teeth without challenges. (E) Participating parents faced more challenges when taking photos of their children’s posterior teeth; only 20% could complete this task without challenges. Time spent on each task are shown in bar graph. In Fig 3D and 3E, the two photos (front teeth and posterior teeth) were taken by the mother of a 4-year-old child using the AICaries application, with no challenges. https://doi.org/10.1371/journal.pdig.0000046.g003

The results of the 7 tasks we assessed in the Step 1 usability test are illustrated in Fig 3. All participating parents (n = 10) were able to locate the app icon on the study cellphone with no challenge; 90% of them could navigate the app interface with no challenges. In the caries risk assessment, 40% of the parents completed the risk assessment without challenges, 40% with cosmetic challenges, and 20% with moderate challenges. For taking photos using the AICaries app, 40% of the parents took photos of their children’s front teeth without challenges. However, parents faced more challenges when taking photos of their children’s posterior teeth; only 20% could complete this task without challenges.

When assessing the time spent on each task (results seen Fig 3), the longest time was spent for completing the caries risk assessment task, with a range of 3–15 minutes. All other tasks, including taking teeth photos with the AICaries app, took less than 1 minute.

The majority (78.6%, 22/28) of the participating parents were able to obtain their children’s teeth photos with satisfactory quality required for oral disease detection (Table 2). In the step 1 usability test, 8 out of 10 parents were able to take photos of their children’ teeth (anterior and posterior teeth) using the App. A total of 106 photos were taken by 80% of study subjects (n = 8) with an average of 10.6 (SD 13.3); out of the total of 106 photos, 70 photos were clear, 56 photos were suitable for caries AI assessment. Among the total 106 photos, 38 (38%) photos were for front teeth, 23 photos were clear, and 19 photos of these clear photos considered diagnostic photos (82.6%). Examples of teeth photos taken by the parents are illustrated in Fig 3. In Fig 3D and 3E, the two photos were taken by the mother of a 4-year-old child using the AICaries application, with no challenges.

**Fig 4.**
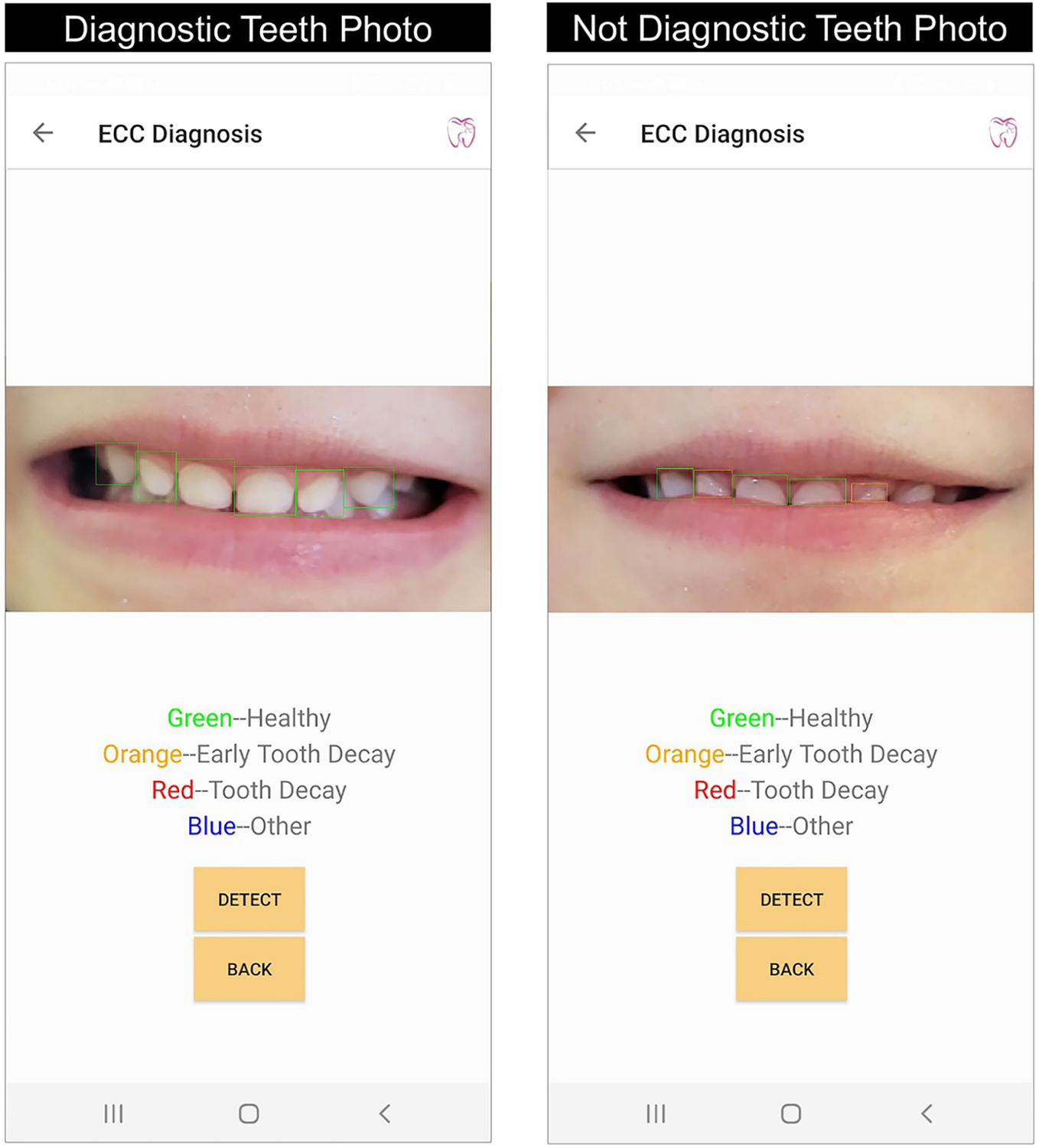
Example of diagnostic and non-diagnostic front teeth photos taken by parents. Examples of a child’s front teeth photo taken by their parents using the AICaries app. The image on the left is clear and diagnostic by including all anatomic structures of the upper front teeth and had a correct diagnosis by AICaries algorithm (all upper front teeth are healthy without tooth decay, marked with green boxes). The image on the right is clear but not diagnostic because the lower lip partially covers the anatomic structure of the upper front teeth. Due to the incomplete tooth picture, AI algorithm did not make correct caries screening for all upper front teeth, two healthy teeth were marked as “Early Tooth Decay” with orange boxes. https://doi.org/10.1371/journal.pdig.0000046.g004

In the step 2 field testing, 89% of the participating parents (25 out of 28) took photos for their children’ teeth (anterior and posterior teeth) using the AICaries App, see Table 2. A total of 334 photos were taken by participating parents, with an average 11.9±12.0 teeth photos per parent; among these photos, 52% (174) were clear, 45% (149) were diagnostic for caries assessment. Furthermore, the majority of the teeth photos taken by parents were front teeth photos (90.7%). Worth noting, 78.6% (22/28) of the parents generated at least 1 diagnostic photo for their children’s front teeth, and no significant differences were seen in terms of their capability of taking diagnostic photos between step 1 and step 2 tests (p = 0.05). Fig 4 shows an example of children’s front teeth photos taken by their parents using the AICaries app. The image on the left is clear and diagnostic by including all anatomic structures of the upper front teeth. The image on the right is clear but not diagnostic because the lower lip partially covers the anatomic structure of the upper front teeth.

### Usability of AICaries in the field

**Fig 5.**
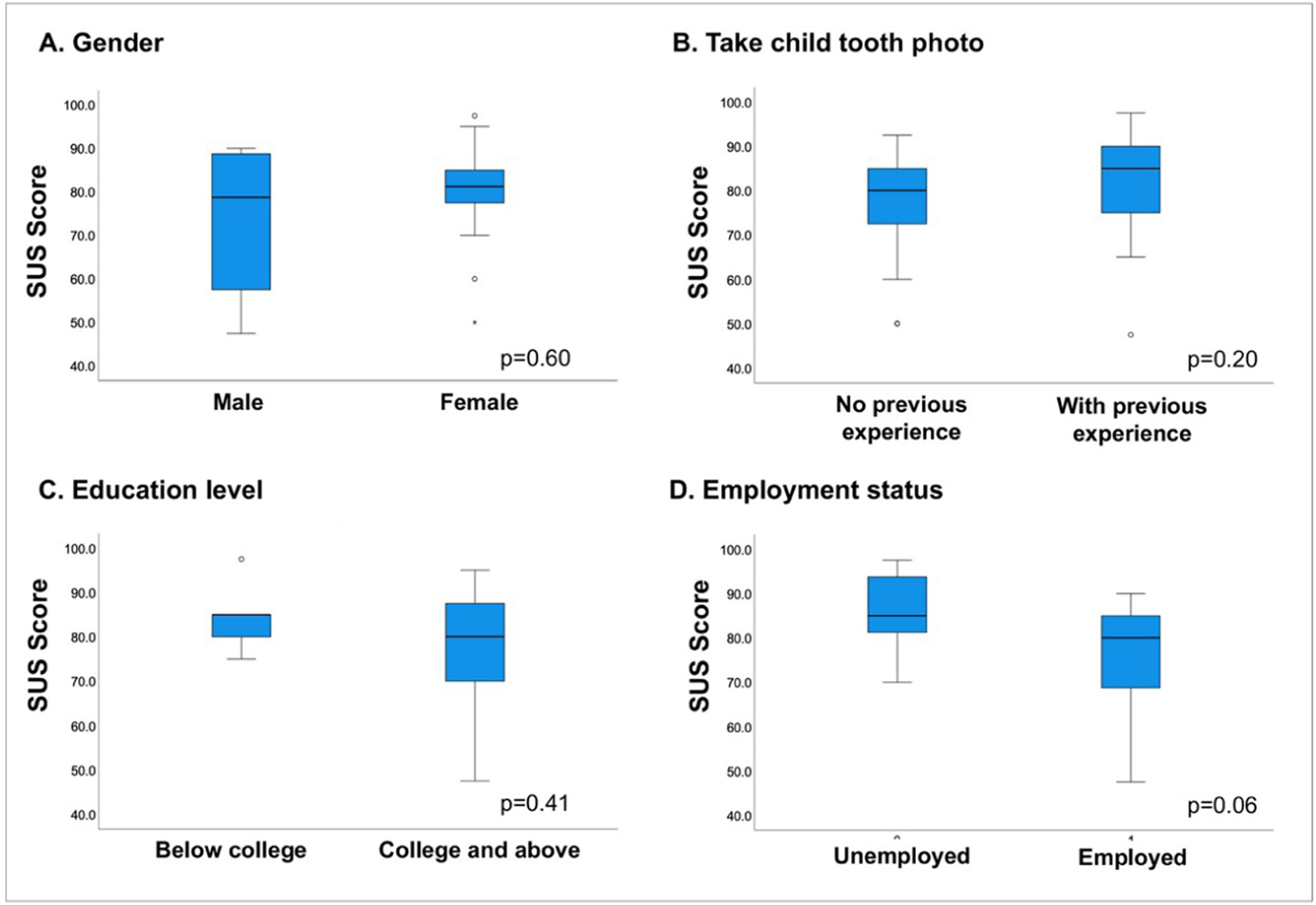
System usability score for AICaries app (Step 2 unmoderated field testing). No significant difference were found between male and female (A), parents who had or did not have previous experience in taking photos for their children’ teeth (B), participants who had equal or more than college and below college education (C), and parents with different employment status (D). n = 28. https://doi.org/10.1371/journal.pdig.0000046.g005

**Fig 6.**
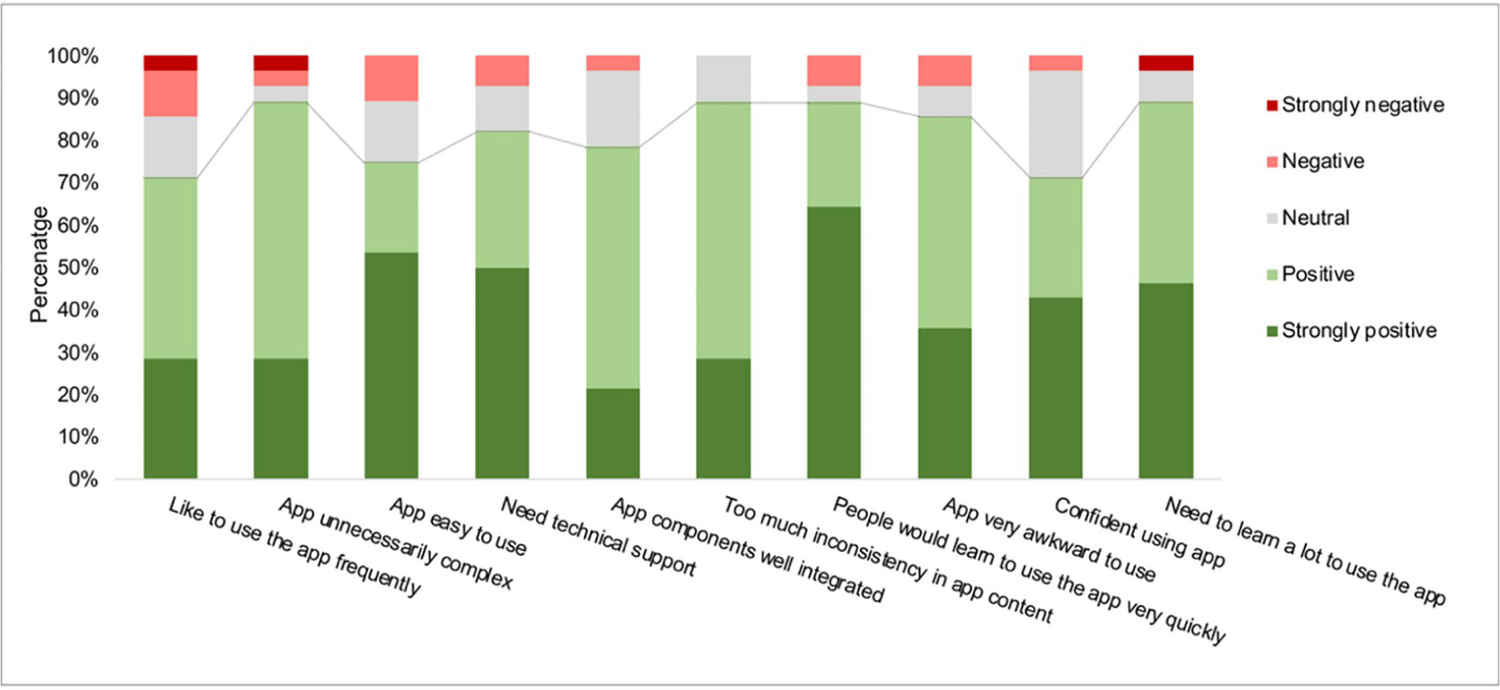
Feedback to system usability scoring items (Step 2 unmoderated field testing). The response to each item of the System Usability Scoring is converted to a scale from strongly negative to strongly positive. Across the ten items, the responses from more than 70% of the participants are positive (positive and strongly positive), indicating the well-perceived usability of AICaries app. For example, 70% of the participants like to use the app frequently and feel confident using the app; less than 10% of the participants feel they need technical support in order to use the app. https://doi.org/10.1371/journal.pdig.0000046.g006

The SUS score for AICaries app had values of 78.4 ± 13.4 (mean ± SD). We found statistically no significant differences in SUS score for gender, educational level, and employment status (p<0.05). Interestingly, there was also no significant difference between parents who had or did not have previous experience in taking photos for their children’ teeth (results seen Fig 5). The rating of individual items of SUS is shown in Fig 6. Moreover, the multiple linear regression analysis did not reveal significant association between gender, race, education, previous experience of taking teeth photo (F-value 1.01, p = 0.42, S1 Table). The response to each the SUS items was converted to a scale from strongly negative to strongly positive. Across the ten items, the responses from more than 70% of the participants are positive (positive and strongly positive), indicating the well-perceived usability of AICaries app. For example, 70% of the participants like to use the app frequently and feel confident using the app; less than 10% of the participants feel they need technical support in order to use the app.

The qualitative interviews confirmed the acceptance of AICaries by participating parents. Selected quotes are listed in Table 3.

### Benefits and Challenges of utilizing AICaries to promote oral health in children

**Fig 7.**
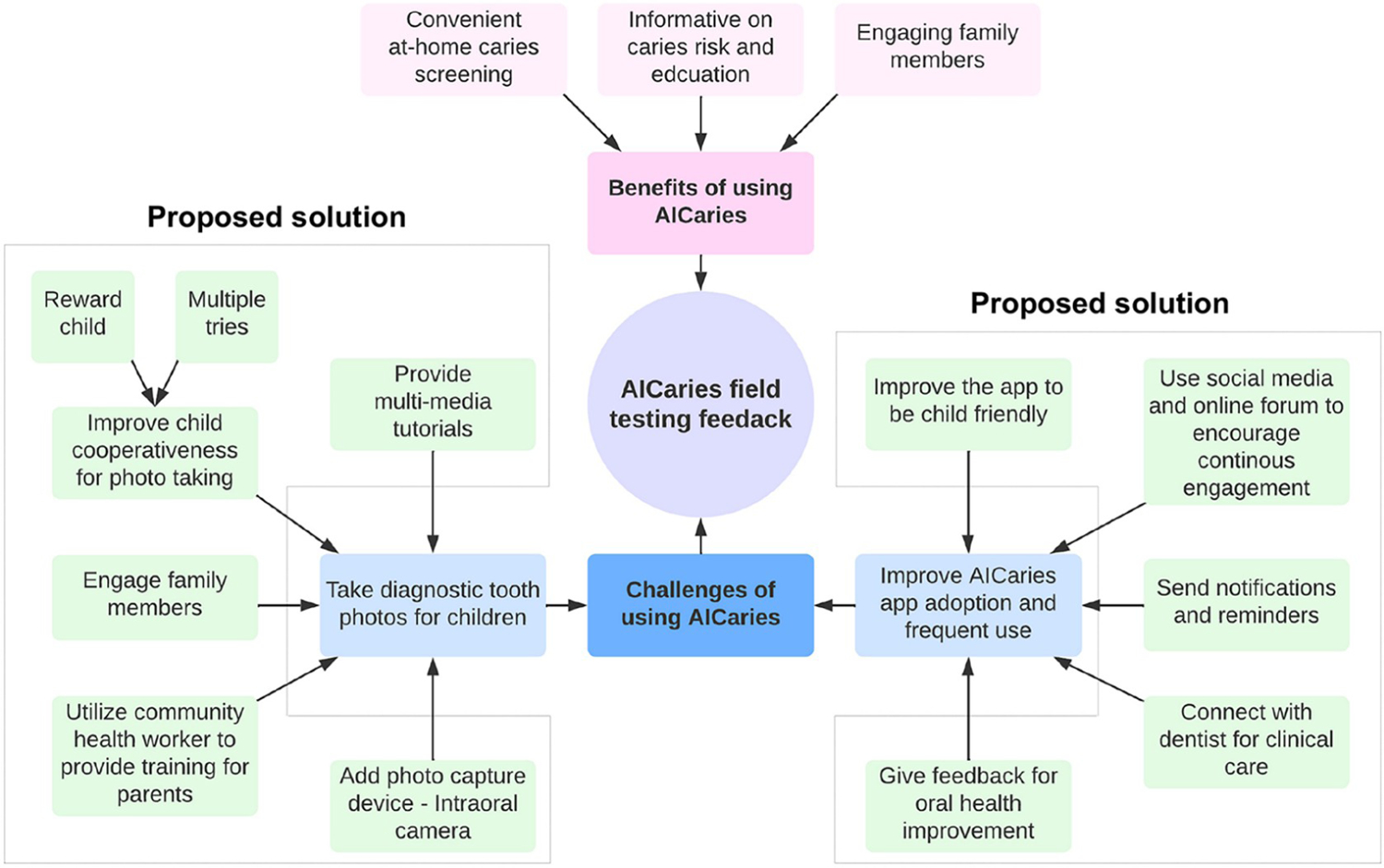
Challenges and Solutions perceived by AICaries app users. https://doi.org/10.1371/journal.pdig.0000046.g007

Fig 7 demonstrates summarized the feedback from participants in Step 2 unmoderated field testing on the benefits perceived and challenges encountered from using the AICaries app and the proposed solutions for solving the challenges for future users. Qualitative analysis indicated various benefits from using the AICaries app, including detecting caries early, helping parents or caregivers maintain and improve their children’s oral health, and encouraging children’s engagement in oral health promotion.

Perceived challenges of using the AICaries app included two main categories: 1) Taking satisfactory tooth photos and 2) Improving AICaries app adoption and continued engagement among users. Although 78% of the parents could take at least 1 diagnostic tooth photo for their children, participants expressed their desire for better-taking photos. Several solutions were proposed to overcome this challenge, including providing multi-media tutorials of taking teeth photos for children using a smartphone, improving child cooperativeness by rewards and multiple tries, engage multiple family members for photo taking. Innovative suggestions including engaging community health workers to assist and train parents and incorporating photo capture devices such as an intraoral camera to enhance intraoral photo taking. Proposed solutions to promoting app engagement were using social media and online forums for promoting community awareness and app engagement, sending notifications and reminders, providing feedback for oral health improvement, and providing clinic resources via connecting patients with dental clinics for needed treatment.

## Discussion

The current study evaluated the usability, acceptability, and feasibility of an innovative smartphone app (AICaries) to be used by parents or caregivers to monitor and improve their children’s oral health. Our study results indicated the AICaries app is well-accepted among the participating parents.

First, the usability of AICaries was assessed by SUS. The usability of any technical product needs to be assessed in terms of how and for what purposes it can be used. The ISO 9241–11 Ergonomics of human-system interaction [25] provides a framework for understanding the concept of usability, and recommends that measures of usability should include: a) effectiveness (the ability of users to complete tasks using the tool, and the quality of the product of these tasks), b) efficiency (the level of resource utilized in accomplishing tasks), and c) satisfaction (users’ reactions to using the tool). The SUS is a reliable widely used tool to assess the usability of technical products. A SUS score above 68 would be considered above average and anything below 68 is below average. The participants in AICaries usability test gave an average SUS score of 78.4, reflecting the fact that AICaries is well-accepted by parents with young children as a tool for at-home dental caries detection, at-home caries risk assessment, and oral health knowledge improvement.

Second, with regards to AICaries acceptability, interviews with 28 parents who participated in the step 2 field testing indicated that the app was viewed favorably in terms of overall quality and functionality. The overall acceptance is reflected in feedback on 3 main themes: (a) participants found the AICaries app is a resourceful tool for improving oral health. Dental caries is a multifactorial disease, and its risk is associated with diet, oral hygiene practice, oral microbial, and host factors. Participants expressed that AICaris empowered them for caries risk assessment which could ultimately help them improve their children’s and their oral health. Nearly all (96.4%, 27/28) participants reported that they would recommend the app to their friends and family members.

(b) a practical tool for at-home caries detection. U.S. national surveys have shown that low-income and minority children are disproportionately affected by ECC and experience limited oral health care access [10,26]. US preschool children from low socioeconomic status (SES) families are much less likely to have at least one dental visit than children from high SES families, despite experiencing higher need [10,26]. To address these oral health disparities, we need to make oral disease screening services easily accessible to patients regardless of social, economic and geographic factors, such as via mHealth tools. Currently, patients can monitor their blood pressure via at-home blood pressure devices; patients can monitor their blood glucose via an at-home glucose meter; patients can even monitor their heart rate and rhythm from wearing devices such as a smartwatch [16]. In contrast, when it comes to monitoring oral health, other than routinely visiting dental professionals, patients have no effective at-home devices to monitor their oral health conditions [16].

AI is significantly changing healthcare services, and it makes the lives of patients and healthcare providers easier by performing tasks in less time and less cost. For example, AI has been used to analyze ECG, EEG, or X-ray images for disease early detection [27]. AI technology has also been used to assist early detection of Rheumatoid Arthritis (RA) [28]. However, modern dentistry has not employed AI imaging technology for caries detection. To our knowledge, the AICaries is a novel application of using this technology in dentistry. Using AICaries, parents can use their regular smartphones to take photos of their children’s teeth and detect ECC aided by AICaries, enabling them to actively seek treatment for their children at early and reversible stages.

(c) Useful educational resource for caries prevention. Current community-based caries education utilizes a one-on-one approach. The oral health education is delivered by dental professionals, such as dentist, hygienist, on the chairside. For families with limited access to care, a community-centered approach that could reach each individual is needed. Using AICaries, parents can also obtain essential knowledge on reducing their children’s caries risk.

Third, regarding the feasibility of AICaries, the metrics of taking photo metrics and feedback from the parents demonstrated the ease-of-use feature of the AICaries. Worth noting, in Step 2 field testing, other than the visual guides on the AICaries app, study team did not provide any additional training for parents on how to use cameras and how to take children’s teeth photos. With this condition, remarkably, 78.6% of the parents in step 2 took children’s photos that passed quality check and were suitable for caries diagnosis by the App.

We recognize that taking a child’s photo with satisfactory quality depends on children’s age, personality, cooperative abilities, and parents’ interest, which are the main reasons for the remaining parents (21.4%) who could not complete this task successfully. To improve the feasibility of the AICaries, parents suggested community health worker facilitated training, which is a remarkable suggestion. Community health workers (CHWs) are mediators linking the local health system and the community with varying degrees of integration into the national health system [29]. Consequently, CHWs play a critical role in grassroots healthcare and are essential for achieving the health-related Sustainable Development Goals [30]. In addition, the CHWs have crucial roles in the qualitative assessment of the dental health aides in remote settings to improve the dental care for children living in these areas. For instance, Indigenous health workers (IHWs) have an essential role in promoting oral health during pregnancy, as there are training programs available for non-dental health professionals to help them promote oral health throughout the prenatal period. Also, IHWs can use available oral health screening tools for women during the prenatal period; they have the potential to play a crucial role in ‘driving’ screening and education of maternal oral health, especially when there is adequate organizational support [31]. Involving CHWs in training parents utilizing technology devices in caries screening would provide innovative and significant reform in community oral health prevention and close the digital divide associated with financial status.

The following limitations need to be considered when interpreting our study results: 1) The study was only conducted in one US city. Thus, the generalizability of our study results to other populations is limited. 2) Only participants with Android devices were enrolled, which might overestimate or underestimate the acceptance among other smartphone platform users. 3) Although all participating parent-child dyads were from low-income families, 66% of the parents in step 2 unmoderated field testing had college-level education; the percentage is higher than the average Americans whose income level is at or lower than the FPL. One reason is that a 36% of the participants in Step 2 were refugees or immigrants born outside of the US, in which situation, their family income is limited despite having a higher-level education background. 4) Lack of comparison to chair-side caries diagnosis by a clinician (gold standard). The ultimate goal of the AICaries app is to achieve at-home caries screening using teeth images taken by the parents or caregivers. As the current study is the first step to assessing the usability, feasibility, and acceptance, future design should incorporate testing the validity of the AICaries and compare the sensitivity and specificity of the AICaries app in detecting caries to the clinicians.

As we are continually improving the function and usability of AICaries smartphone app, our future direction will focus on developing a strategy to encourage parents’ frequent use of the AICaries in their natural environment, which is vital to achieving population screening for dental caries in a young age. Moreover, future clinical trials are needed to evaluate the real-world impact of using this innovative smartphone app on the early detection and prevention of ECC among low-income children.

## Conclusions

Using AICaries, many parents are able to use their regular smartphones to take photos of their children’s teeth for detection of ECC aided by AICaries. Potentially, doing so will promote pediatric dental treatment at an early and reversible stage of ECC. Using AICaries, parents can also obtain essential knowledge on reducing their children’s caries risk. Data from this study will support future clinical trial that evaluates the real-world impact of using this innovative smartphone app on early detection and prevention of ECC among low-income children.

## Supplementary Material

Table S1 S1 Table. Multiple linear regression with System Usability Score as the independent variables. (DOCX)

## Figures and Tables

**Table 1.** Demographic-socioeconomic-behavior information of participants.

		Step 1 moderated usability test (n = 10)	Step 2 unmoderated field testing (n = 32)
Age (Year)		30±6	34±5
Gender (Female)		9 (90%)	28 (88%)
Race	Black	2 (20%)	10 (31%)
	White	6 (60%)	17 (53%)
	Other	2 (20%)	5 (16%)
Ethnicity (Hispanic)		20%	9%
Employed		50%	69%
Marital status	Married	6 (60%)	25 (78%)
	Other	4 (40%)	7 (22%)
Education	≧College	3 (30%)	21 (66%)
	High school	5 (50%)	9 (28%)
	Middle school	2 (20%)	2 (6%)
Child daycare attendance (Yes)		2 (20%)	11 (34%)
Used medical care related app (Yes)		9 (90%)	31 (97%)
Use dental care related Apps (Yes)		0 (0%)	0 (0%)
Past experience of using cell phone to take teeth photos (Yes)		6 (60%)	19 (59%)

Values are presented in mean (SD) and n (%).

* p<0.05

https://doi.org/10.1371/journal.pdig.0000046.t001

**Table 2.** Intraoral photos taken by participants.

		Step 1 moderated usability test (n = 10)	Step 2 unmoderated field testing (n = 28)
**All teeth photos (Front + posterior)**	Total photos (number)	10.6±13.3	11.9±12.0
	Clear photos (number)	7.0±10.7	6.2±5.4
	Diagnostic photos (number)	5.6 ± 9.1	5.3±4.7
	Parents took ≥ 1 diagnostic photos	50% (5)	78.6% (22)
**Front teeth photos**	Total photos (number)	3.8±3.2	10.8±11.8
	Clear photos (number)	2.3±2.4	5.8±5.3
	Diagnostic photos (number)	1.9±2.2	5.0±4.7
	Parents took ≥ 1 diagnostic photos	50% (5)	78.6% (22)

Values are presented in mean (SD).

* p<0.05.

https://doi.org/10.1371/journal.pdig.0000046.t002

**Table 3.** Illustrative Quotations from participants on the acceptance of AICaries.

Acceptance	Illustrative Quotations
**Resourceful tool for improving oral health**	“The app is very convenient to use, a lot of information regarding the risk factors, a lot of videos, helps to improve the oral health. So, in my opinion, it’s a really good app.”
**At-home caries detection**	“I can use it instead of calling my dentist, it’ll tell you if there’s a cavity or something, I really think a lot of people benefit from that.”
**Tool for risk assessment and caries prevention**	“It could be a good prevention tool, especially for those who have a limited access to dental care.” “It is very helpful in measuring Caries risk assessment, helping in the modification of child diet.”
**Ease of use**	“I felt like it was very easy for parents to use and also the kids enjoyed using it as well. So, it was something that we enjoy together.”

https://doi.org/10.1371/journal.pdig.0000046.t003

## Data Availability

All data are in the manuscript and/or supporting information files.
