# Supplementary material for: Artificial intelligence-powered smartphone application, AICaries, improves at-home dental caries screening in children: Moderated and unmoderated usability test
Source: PLOS Digit Health. 2022 Jun 2;1(6):e0000046. doi: 10.1371/journal.pdig.0000046 (PMC9645586; doi:10.1371/journal.pdig.0000046)
Supplement: S1 Table — (DOCX) [file pdig.0000046.s001.docx]

**S1 Table. Multiple linear regression with System Usability Score as the independent variables**

| Parameter | Estimate | SE | T Value | Significance |
| --- | --- | --- | --- | --- |
| Intercept | 75.72 | 9.18 | 8.25 | <.001 |
| Race | -2.19 | 5.94 | -0.37 | 0.72 |
| Female | 6.31 | 6.18 | 1.02 | 0.32 |
| Education | -4.32 | 6.69 | -0.65 | 0.52 |
| Previous experience of taking child tooth photo | 5.88 | 5.50 | 1.07 | 0.30 |

n=28
